# Adult body height and age-related macular degeneration in healthy individuals: A nationwide population-based survey from Korea

**DOI:** 10.1371/journal.pone.0232593

**Published:** 2020-05-01

**Authors:** In Cheol Hwang, Jeong Hun Bae, Joon Mo Kim, Jung Min Lee, Quan Dong Nguyen

**Affiliations:** 1 Department of Family Medicine, Gil Medical Center, Gachon University College of Medicine, Incheon, Republic of Korea; 2 Department of Ophthalmology, Kangbuk Samsung Hospital, Sungkyunkwan University School of Medicine, Seoul, Republic of Korea; 3 Byers Eye Institute, Stanford University School of Medicine, Palo Alto, California, United States of America; National Yang-Ming University Hospital, TAIWAN

## Abstract

We sought to evaluate the relationship between adult body height and risk of age-related macular degeneration (AMD) among healthy Koreans using nationwide population-based data. We analyzed data derived from the Korea National Health and Nutrition Examination Survey 2008–2011. Participants over 40 years of age were included in the sample after excluding individuals with systemic comorbidities or missing relevant data. The presence and severity of AMD were graded using fundus photographs. The relationship between body height and risk of AMD was determined using multiple logistic regression analyses. Among a total of 8,435 participants, 544 (6.45%) had AMD: 502 (5.95%) with early AMD and 42 (0.5%) with late AMD. In multivariate-adjusted analyses, taller body height was significantly associated with a lower prevalence of AMD (odds ratio [OR], 0.89; 95% confidence interval [CI], 0.81–0.99), while body mass index (BMI) was not associated with AMD. An inverse association between body height and risk of AMD was observed most frequently in participants under 65 years of age (OR, 0.81; 95% CI, 0.70–0.94). Furthermore, body height showed an inverse association with risk of AMD among obese participants (BMI ≥25.0 kg/m^2^) (OR, 0.75; 95% CI, 0.60–0.93). Subgroup analysis by AMD type disclosed a significant inverse association between body height and early AMD (OR, 0.87; 95% CI, 0.79–0.97) but not late AMD. Our results suggest that shorter body height is independently associated with increased risk of AMD, especially early AMD, in a dose-response manner in people who are obese or under 65 years of age.

## Introduction

Age-related macular degeneration (AMD) is a major cause of blindness worldwide, affecting primarily those 40 years of age or older in developed countries [1]. Approximately 10% of patients with early AMD progress to late AMD, which is characterized by geographic atrophy of the retinal pigment epithelium (RPE) and photoreceptors or choroidal neovascularization, which can lead to severe vision loss [1]. Although anti-angiogenic therapies have partial efficacy in neovascular AMD, no treatment is effective for the majority of AMD patients [2]. Studies to identify risk factors for AMD are important to prevent the development and progression of this intractable disease.

Efforts are under way to determine risk factors for AMD, and several studies have suggested that anthropometric factors such as body mass index (BMI) and waist circumference play roles in the development of AMD. Overweight or obesity defined by BMI have been reported to be associated with AMD, but the results of such studies are inconsistent [3]. Some have shown that higher BMI may increase the risk of AMD, while others have failed to detect this relationship [4–7]. In fact, BMI does not represent all aspects of obesity [8], and is considered a proxy of current physical condition, whereas body height, an element in BMI calculation, is determined by interactions among genetic predisposition, nutritional status, and socioeconomic environment during childhood and adolescence. Thus, body height may be a good anthropometric indicator reflecting both genetic and environmental influences on certain conditions.

There are robust epidemiologic findings regarding the influence of body height as a risk factor for several diseases. Previous studies reported that the risks of stroke and coronary artery disease are significantly associated with short body height [9,10]. A recent Japanese study has also indicated that body height is inversely related to carotid atherosclerosis among overweight men [11]. As the atherosclerotic process is similar to drusen deposition and likely affects choroidal blood flow and the pathophysiology of the RPE, atherosclerotic vascular diseases and AMD may share similarities in pathogenesis as well as risk factors [12,13].

Unlike cardiovascular disease, the relationship between body height and AMD has not been sufficiently evaluated. In this study, we investigate the relationship between body height and risk of AMD, using data from a nationwide cross-sectional cohort of the Korean population.

## Materials and methods

### Data source and study participants

We analyzed data obtained from the Korean National Health and Nutrition Examination Survey (KNHANES) 2008–2011, which is a nationwide population-based survey conducted periodically by the Korean Ministry of Health and Welfare. Detailed information regarding survey design has been published elsewhere [14]. Individuals were randomly selected through a stratified, multistage, probability-sampling design according to sampling units based on age groups using household registries and economic status, sex, and geographical area. In 2008–2011, a total of 46,777 individuals older than 1 year of age were included in the survey, and of these, 37,753 individuals participated in the health examination survey (overall response rate, 80.7%).

All KNHANES interviews and examinations were conducted in specially designed and equipped mobile centers throughout the country. All participants provided written informed consent to participate in the study, and the KNHANES was conducted according to the guidelines put forth in the Declaration of Helsinki. The study protocol was approved by the Institutional Review Board of the Korea Center for Disease Control and Prevention (IRB No: 2008-04EXP-01-C, 2009-01CON-03-2C, 2010-02CON-21-C). As KNHANES data are anonymous and publicly available on the KNHANES website, this study was exempt from requirements for approval by the Institutional Review Board of the Kangbuk Samsung Hospital.

We identified 16,014 eligible participants 40 years of age or older who underwent ophthalmic examinations. Participants were excluded if they had any of the following comorbidities: (i) history of malignancy, (ii) cardiovascular disease, (iii) diabetes mellitus or taking glucose-lowering agents, (iv) hypertension or taking antihypertensive medications, and (v) dyslipidemia or taking antihyperlipidemic agents. A history of disease was defined by physician diagnosis. Of the remaining 9,287 who had no apparent comorbidities, 852 participants without a gradable fundus photograph of either eye were also excluded from the study. Therefore, a final dataset of 8,435 participants was included in analysis.

### Data collection and variable definitions

The KNHANES consists of three components: the Health Interview Survey, the Health Examination Survey, and the Nutrition Survey. The health interviews and health examinations were performed during a single day by trained medical staff and interviewers at mobile examination centers. One week after the health surveys, dieticians visited the homes of participants and conducted nutrition surveys [15]. Information on age, sex, health behaviors (smoking history and physical activity), history of physician-diagnosed diseases, and current medications was collected during the interview. Physical activity was classified using the International Physical Activity Questionnaire short-form scoring protocol, and each participant’s physical activity was classified as either “regular exercise” (moderate-intensity activity more than five times per week for at least 30 minutes per session or vigorous activity more than three times per week for at least 20 minutes per session), or “other” [16]. A current smoker was defined as an individual who smoked cigarettes at the time of the interview. Alcohol consumption was categorized as “at-risk drinking”, which was defined as more than seven drinks (men) or five drinks (women) in one sitting more than two days per week, or “other”.

After each interview, body height and weight were measured on a standard scale with the participants wearing light clothing and no shoes, and BMI was calculated as weight (kg) divided by height squared (m^2^). Obesity was defined as BMI ≥25.0 kg/m^2^ according to WHO criteria for the Asia-Pacific region, and participants were divided into two groups according to BMI; a non-obese group with BMI <25.0 kg/m^2^ and an obese group with BMI ≥25.0 kg/m^2^ [17].

### Ophthalmologic examinations

All participants underwent detailed ocular examinations, including measurement of visual acuity and intraocular pressure, autorefraction, slit-lamp biomicroscopy, and fundus photography. Certified ophthalmologists performed all ocular examinations, and the Epidemiologic Survey Committee of the Korean Ophthalmologic Society verified the quality of the ophthalmic surveys [18]. The spherical equivalent was calculated as the spherical value plus half of the cylindrical value. Digital fundus photographs centered on the fovea (Diabetic Retinopathy Study standard field 2) were taken with a non-mydriatic fundus camera (TRC-NW6S, Topcon, Tokyo, Japan; Nikon D-80, Nikon, Tokyo, Japan) under physiological mydriasis. Fundus images were graded in two steps (preliminary and detailed grades) using the International Age-related Maculopathy Epidemiological Study Group protocol [19]. Detailed grading was conducted independently by nine retina specialists entrusted by the Korean Ophthalmologic Society, and final grading was based on detailed grading. If there was any disagreement between the preliminary and detailed grades, one specialist made the final grade. The interrater reliability for AMD grading was 90.2% and 90.7% in 2008, 92.4% and 93.3% in 2009, 94.1% and 95.0% in 2010, and 96.2% and 96.6% in 2011 (right eye and left eye, respectively).

Early AMD was identified if the fundus photograph met either of the following criteria: (1) the presence of soft indistinct drusen or reticular drusen, or (2) the presence of hard or soft distinct drusen with a pigmentary abnormality, such as RPE depigmentation or increased pigmentation, in the absence of signs of late AMD. Late AMD included the presence of signs of wet AMD or geographic atrophy. Wet AMD was defined as RPE detachment or serous detachment of the sensory retina, subretinal or sub-RPE hemorrhage, and subretinal fibrous scars. Geographic atrophy was defined as a discrete circular area (175 μm in diameter) of retinal depigmentation with visible choroidal vessels, in the absence of signs of wet AMD. If both eyes were gradable, the eye with more severe conditions (“worse eye”) was used for the AMD classification of a participant.

### Statistical analysis

All analyses were performed using Stata/SE software version 9.2 (Stata Corp., College Station, TX). Descriptive statistics are presented as mean ± standard deviation or percentage. Differences in the characteristics of study participants according to AMD status were evaluated using a Wilcoxon rank-sum test or chi-square test. Body mass index was assessed using both continuous and categorical variables according to the Asia-Pacific classification, and body height was analyzed as both continuous and categorical variables. A multiple logistic regression model was used to evaluate the relationships between body height and the presence or stage of AMD, adjusted for potential confounding factors that included age and health-related behaviors. We calculated odds ratios (ORs) and 95% confidence intervals (CIs) per 10-cm increase in height as trends across the category of height. All statistical tests were two-tailed, and results with p < 0.05 were considered statistically significant.

## Results

Comparisons between participants with and without AMD grading are shown in S1 Table. Of the 8,435 participants (4,861 women, 57.6%), 544 (6.45%) had AMD; 502 (5.95%) had early AMD and 42 (0.50%) had late AMD. The prevalence of AMD did not differ by sex. Table 1 shows the characteristics of study participants according to the presence of AMD. Participants with AMD tended to be older and to be at-risk drinkers, and were less likely to exercise regularly compared to those without AMD (all p < 0.05). Furthermore, participants with AMD were more likely to have hyperopic refractive error (>+0.5 D) and previous cataract surgery in their eyes (both p < 0.001). The proportions of participants who had higher BMI (≥25.0 kg/m^2^) and taller body height (≥170 cm) were greater in the no AMD group (both p < 0.001).

**Table 1:** Baseline characteristics of study participants with and without AMD.

	No AMD (*n* = 7,891)	AMD (*n* = 544)	*p*-value
Age, years	53.6 ± 10.8	64.3 ± 10.5	<0.001^a^
40–49, *n* (%)	3,436 (43.5)	53 (9.7)	<0.001^b^
50–59, *n* (%)	2,333 (29.6)	125 (23.0)	
60–69, *n* (%)	1,250 (15.8)	178 (32.7)	
≥70, *n* (%)	872 (11.1)	188 (34.6)	
Women, *n* (%)	4,548 (57.6)	313 (57.5)	0.964^b^
Health-related behaviors			
Current smoker, *n* (%)	1,557 (19.7)	100 (18.4)	0.444^b^
At-risk drinking, *n* (%)	409 (5.2)	39 (7.2)	0.046^b^
Regular exercise, *n* (%)	1,988 (25.7)	102 (19.4)	0.001^b^
Hyperopia, *n* (%)	4,484 (56.8)	423 (77.8)	<0.001^b^
Cataract surgery, *n* (%)	169 (2.1)	37 (6.8)	<0.001^b^
Body mass index, kg/m^2^	23.5 ± 3.0	22.9 ± 2.9	<0.001^a^
<25.0, *n* (%)	5,632 (71.4)	423 (77.8)	<0.001^b^
≥25.0, *n* (%)	2,259 (28.6)	121 (22.2)	
Body height, cm	161.2 ± 8.8	158.4 ± 9.1	<0.001^a^
<150, *n* (%)	735 (9.3)	100 (18.4)	<0.001^b^
150–159, *n* (%)	2,672 (33.9)	198 (36.4)	
160–169, *n* (%)	2,877 (36.5)	178 (32.7)	
≥170, *n* (%)	1,607 (20.4)	68 (12.5)	

AMD, age-related macular degeneration.

Data are presented as mean ± standard deviation or frequency (%).

^a^Wilcoxon rank-sum test was used for continuous variables.

^b^Chi-square test was used for categorical data.

### Anthropometric parameters and AMD

Results of logistic regression analyses are provided in Table 2. Body height showed a significant and independent inverse association with the presence of AMD, even after adjusting for age, alcohol consumption, physical activity, hyperopia, and cataract surgery (OR, 0.89; 95% CI, 0.81–0.99; p = 0.033). BMI also revealed an inverse association with AMD, but failed to show independent significance after adjusting for age, alcohol consumption, physical activity, hyperopia, and cataract surgery (p = 0.089).

**Table 2:** Factors associated with the presence of AMD.

	OR (95% CI)	*p*-value	OR (95% CI)^a^	*p*-value
Age, per 10 years	2.29 (2.11–2.49)	<0.001		
Women	1.00 (0.83–1.19)	0.964		
Current smoker	0.88 (0.73–1.08)	0.444		
At-risk drinking	1.41 (1.00–1.99)	0.047		
Regular exercise	0.69 (0.56–0.87)	0.001		
Hyperopia	2.66 (2.16–3.27)	<0.001		
Cataract surgery	3.33 (2.31–4.81)	<0.001		
BMI, per 1 kg/m^2^	0.93 (0.90–0.96)	<0.001	0.97 (0.94–1.00)	0.089
Body height, per 10 cm	0.70 (0.64–0.78)	<0.001	0.89 (0.81–0.99)	0.033

AMD, age-related macular degeneration; BMI, body mass index; CI, confidence interval; OR, odds ratio.

^a^Adjusted for age, alcohol consumption, physical activity, hyperopia, and cataract surgery.

**Fig 1:**
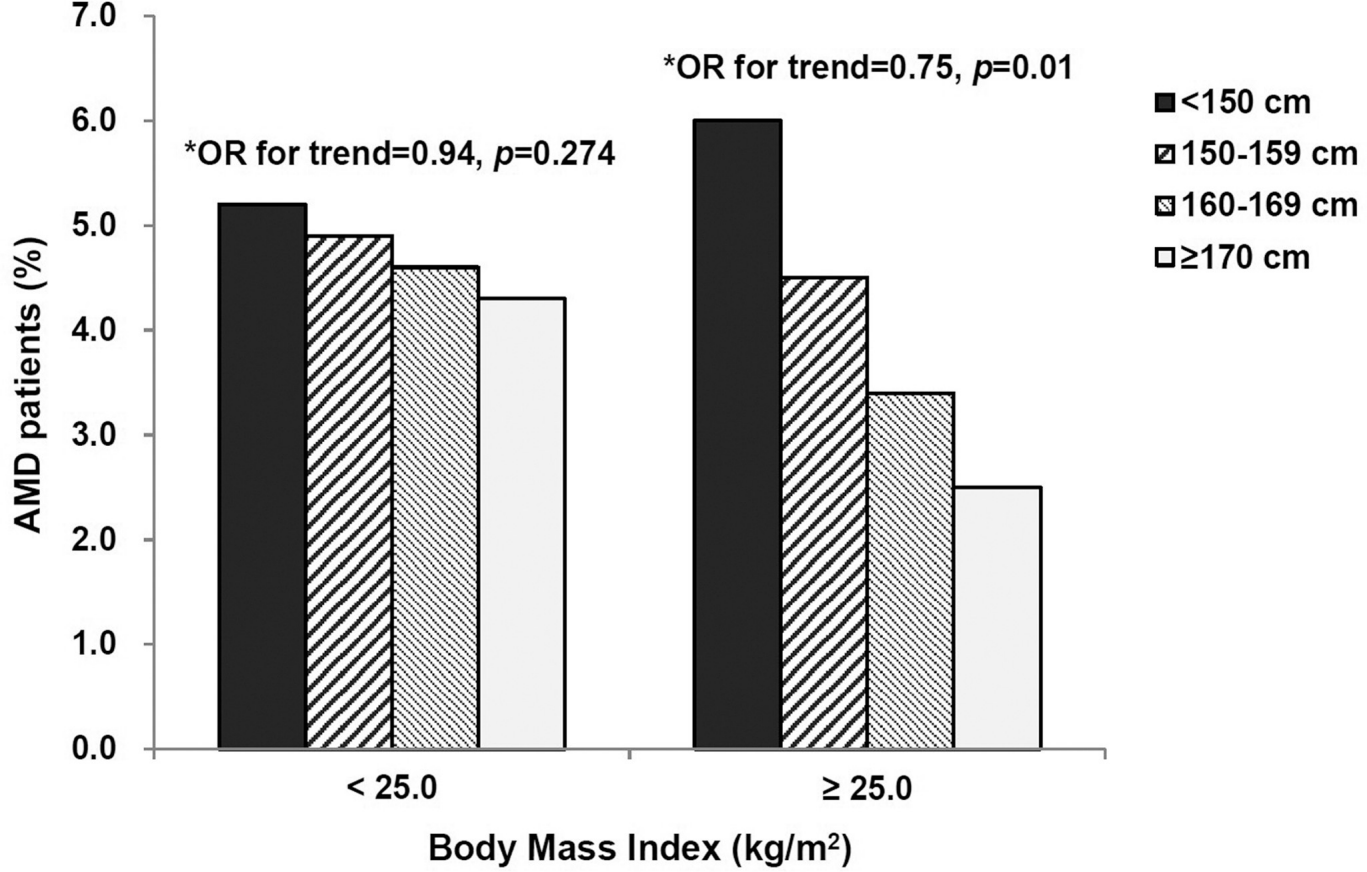
Estimated overall age-related macular degeneration (AMD) prevalence in the non-obese and the obese groups stratified by body height. Obesity is defined as body mass index ≥25.0 kg/m^2^ according to the Asia-Pacific classification. *Adjusted for age, alcohol consumption, physical activity, hyperopia, and cataract surgery.

Fig 1 shows the differences in estimated overall AMD prevalence and the ORs for trend according to body height in the non-obese and the obese groups. There was a significant inverse association between body height and the presence of AMD for obese participants (OR, 0.75; 95% CI, 0.60–0.93; p = 0.01), but not for non-obese participants.

### Body height and AMD by age group

**Fig 2:**
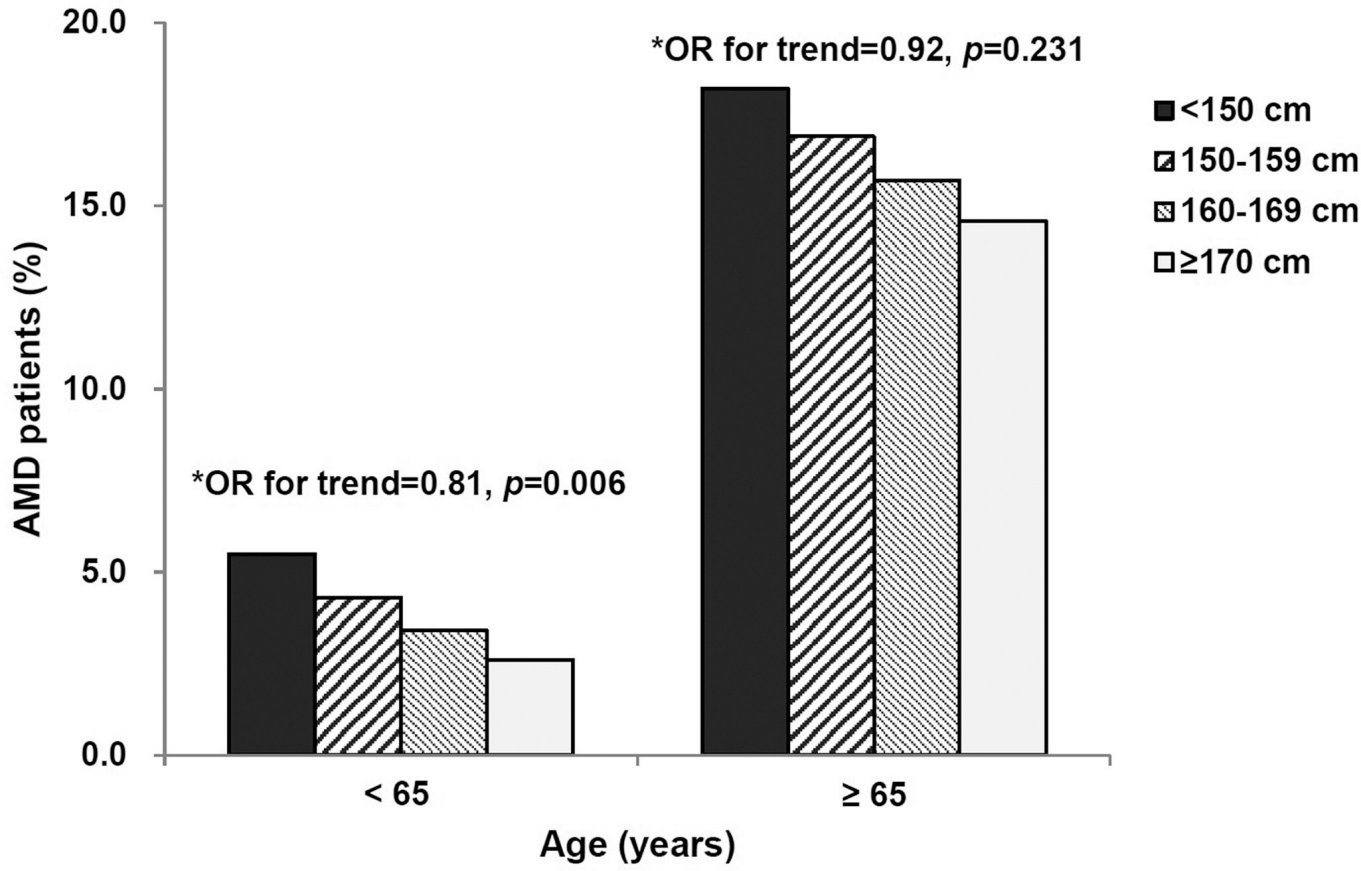
Estimated overall age-related macular degeneration (AMD) prevalence in younger and older age groups stratified by body height. *Adjusted for alcohol consumption, physical activity, hyperopia, and cataract surgery.

We also evaluated the relationships between body height and AMD for different age groups, divided by the age of 65 years (Fig 2). When body height was categorized into four groups, multivariate analyses indicated that body height was inversely associated with having AMD after covariate adjustments including alcohol consumption, physical activity, hyperopia, and cataract surgery only for participants under 65 years of age (OR, 0.81; 95% CI, 0.70–0.94; p = 0.006). Participants in the tallest group (≥170 cm) had significantly lower odds of having AMD compared to those in the shortest group (<150 cm) (OR, 0.78; 95% CI, 0.65–0.95; p = 0.014).

### Body height and AMD by AMD type

Table 3 shows the ORs and 95% CIs for developing early or late AMD according to body height. In contrast to late AMD, shorter body height was significantly associated with the presence of early AMD, and the adjusted OR for early AMD for an increment of body height of 1 cm was 0.99 (95% CI, 0.98–1.00; p = 0.024). In logistic regression analysis using body height, the trends were found to be significant across categories (OR, 0.87; 95% CI, 0.79–0.97; p = 0.014) after adjusting for age, alcohol consumption, physical activity, hyperopia, and cataract surgery in participants with early AMD. The multivariate-adjusted OR for the tallest (≥170 cm) compared with the shortest group of body height (<150 cm) was 0.86 (95% CI, 0.74–0.98; p = 0.028).

**Table 3:** Adjusted odds ratios and 95% confidence intervals for early and late AMD by body height.

	Early AMD			Late AMD		
	*n*	OR (95% CI)^a^	*p*-value	*n*	OR (95% CI)^a^	*p*-value
Body height, per 1 cm	502	0.99 (0.98–1.00)	0.024	42	1.02 (0.98–1.06)	0.268
Body height category, cm						
<150	93	1		7	1	
150–159	188	0.99 (0.74–1.32)	0.938	10	1.05 (0.38–2.89)	0.929
160–169	161	0.94 (0.81–1.09)	0.399	17	1.08 (0.67–1.72)	0.762
≥170	60	0.86 (0.74–0.98)	0.028	8	1.05 (0.69–1.59)	0.812
*p* for trend		0.87 (0.79–0.97)	0.014		1.12 (0.79–1.58)	0.523

AMD, age-related macular degeneration; CI, confidence interval; OR, odds ratio.

^a^Adjusted for age, alcohol consumption, physical activity, hyperopia, and cataract surgery.

## Discussion

The major finding of our study is that adult body height showed a significant and independent inverse association with prevalence of AMD in healthy participants after adjusting for confounding factors. Participants with shorter body height had a significant risk of early AMD, but not for late AMD. This inverse association of body height with AMD was observed particularly among participants aged less than 65 years and obese participants. Furthermore, dose-response analyses revealed a linear relationship between body height and the risk of AMD, suggesting that body height may be a risk factor or predictor for AMD.

Numerous large prospective studies have demonstrated that body height in adulthood has significant relationships with risk of cardiovascular disease and various malignancies [9,20–27]. A large population-based study including more than 16 million Korean participants with a 9-year follow-up period reported that shorter body height was significantly related to higher risks of myocardial infarction, heart failure, stroke, and all-cause death after adjusting for confounding factors, and this inverse association was found in both men and women [21]. Studies in Western countries also revealed that cardiovascular events had an inverse association with body height [9,22–24]. Meanwhile, another study of body height and cancer identified a significant trend of increasing overall cancer risk with increasing body height in a large prospective cohort of 1.2 million middle-aged women in the United Kingdom [27]. Furthermore, the height-associated cancer risk was similar across populations from Europe, North America, Asia, and Australasia and independent of other risk factors [27].

There have been several reports of relationships between body height and morphometric parameters of the eye. Taller subjects were more likely to have larger eyes with longer axial length, lower corneal refractive power, deeper anterior chamber, and longer vitreous cavity [28]. Based on the observation that body height was inversely correlated with the peripheral depth of the anterior chamber, shorter individuals were hypothesized to have a higher risk of angle-closure glaucoma [29]. In addition, the Beijing Eye Study showed that taller body height was associated with higher cerebrospinal fluid pressure and lower trans-lamina cribrosa pressure difference, resulting in a lower prevalence of open-angle glaucoma [30]. A prospective study in the US, which included 17,150 healthy male physicians, found that taller body height was a risk factor for nuclear cataract [31]. However, the association of body height with ocular diseases has been inconsistent among studies. The Beijing Eye Study conducted in 2006 failed to find any relationship between body height and major ocular diseases including glaucoma, cataract, and AMD [32]. This discrepancy might be caused by differences in study methodology and participants.

We demonstrated a significant relationship between body height and risk of AMD. Although the pathophysiologic mechanism underlying this relationship was not explored, several explanations may account for our results. Adult body height is determined by multiple genetic and environmental factors, especially in childhood [33]. There is a close link between genetically determined shorter body height and adverse lipid profiles, which might be implicated in AMD pathogenesis [9,34]. Emerging studies have established that people with a genetic predisposition to taller height are at lower risk of cardiovascular disease, and given that cardiovascular disease and AMD share similarities in pathogenesis and risk factors including susceptibility genes, genetic factors may explain some of the association between body height and AMD [35–38]. Environmental factors throughout childhood, such as poor nutrition and low socioeconomic status, have also been suggested to be associated with short body height in adulthood and therefore risk of AMD [39–41]. In addition, shorter body height is associated with smaller eyes showing altered choroidal hemodynamics, which may play a role in the pathogenesis of AMD [42].

Adult body height showed an inverse association with overall prevalence of AMD in younger participants (age <65 years), but this tendency was attenuated and not significant in older participants (age ≥65 years). The higher prevalence of AMD associated with shorter body height among younger people may be attributed to greater susceptibility to genetic or early environmental factors that determine body height. Given that old age is the most important risk factor in the development of AMD, the impact of body height on AMD prevalence is relatively diminished in older people.

The association between body height and risk of AMD was revealed to be especially significant in early AMD. In fact, different mechanisms are suggested to be involved in the pathogenesis of early and late AMD, and body height may have greater influence during early stages of disease [43]. Despite examining a large population, however, the number of patients with late AMD in our study was not sufficient to detect an association between body height and late AMD. On the other hand, to minimize the effects of bias and confounding, we excluded subjects with systemic comorbidities that might affect body height as well as the risk of AMD. As a result, differences in prevalence of late AMD among groups might have been diluted by the exclusion process. Thus, a larger prospective randomized cohort study is needed so that the effect of body height on late AMD can be adequately evaluated.

The reasons why the inverse association between body height and risk of AMD was restricted to obese participants also warrant discussion. In our study, the prevalence of AMD was not affected by BMI, whereas body height significantly influenced risk of AMD in the obese. Based on our results, shorter body height, rather than excessive body fat, plays a significant role in the development of AMD among obese people. However, some studies have recognized that higher risk of endothelial dysfunction and inflammatory processes caused by obesity may be involved in AMD pathogenesis [44,45]. Compared to taller participants in our study, shorter participants may already have been at higher risk of AMD, and obesity may increase the risk further. Additional studies are needed to confirm the potential combined effect of short body height and obesity in the development of AMD.

Our study has a number of strengths that should be noted. It was a large population-based study with a high response rate, and grading of AMD was performed using a detailed two-step grading system. However, several limitations should be considered when interpreting the results. First, we were unable to infer causal relationships between AMD development and risk factors due to the cross-sectional study design. Second, as individuals with systemic comorbidities or non-gradable fundus photographs were excluded from the study, selection bias may have affected our analyses of risk factors of AMD, and the prevalence of AMD may have been underestimated. In addition, because our study included apparently healthy participants that do not represent the general population, the results must be interpreted with caution and confirmed in future studies. Third, environmental factors such as health and nutritional status in childhood that could affect adult height were not investigated. Despite these limitations, our findings are informative. We identified a significant inverse association between body height and prevalence of AMD. Our results provide further evidence that body weight control in obese people with shorter body height may reduce the risk of AMD. Larger prospective cohort studies to confirm this relationship are warranted.

In conclusion, shorter body height was significantly related to higher prevalence of AMD in healthy individuals after adjusting for confounding factors. In a subgroup analysis, we found that shorter body height significantly increased the risk of early AMD, especially in individuals who were obese or under 65 years of age.

## Supporting information

S1 Table Comparisons between participants with and without AMD grading. (PDF)

S1 Fig Scatterplots showing the relationship between refractive error and body height in participants with and without AMD. (PDF)
