# Supplementary material for: Adult body height and age-related macular degeneration in healthy individuals: A nationwide population-based survey from Korea
Source: PLoS One. 2020 May 1;15(5):e0232593. doi: 10.1371/journal.pone.0232593 (PMC7194362; doi:10.1371/journal.pone.0232593)
Supplement: S1 Table — (PDF) [file pone.0232593.s001.pdf]

S1 Table. Comparisons between participants with and without AMD grading.

	Gradable (n = 8,435)	Non-gradable (n = 852)	p-value
Age, years	54.3 ± 11.1	63.3 ± 13.6	<0.001[a]
40−49, *n* (%)	3,489 (41.4)	186 (21.8)	<0.001[b]
50−59, *n* (%)	2,458 (29.1)	153 (18.0)	
60−69, *n* (%)	1,428 (16.9)	199 (23.4)	
≥70, *n* (%)	1,060 (12.6)	314 (36.9)	
Women, *n* (%)	4,681 (57.6)	465 (54.6)	0.086[b]
Health-related behaviors			
Current smoker, *n* (%)	1,657 (19.6)	191 (22.4)	0.053[b]
At-risk drinking, *n* (%)	448 (5.3)	48 (5.6)	0.690[b]
Regular exercise, *n* (%)	2,090 (25.3)	146 (18.7)	<0.001[b]
Hyperopia, *n* (%)	4,907 (58.2)	558 (65.5)	<0.001[b]
Cataract surgery, *n* (%)	206 (2.4)	37 (4.3)	0.001[b]
Body mass index, kg/m^2	23.4 ± 3.0	23.2 ± 3.2	0.014[a]
<25.0, n (%)	6,055 (71.8)	614 (72.1)	0.862[b]
≥25.0, n (%)	2,380 (28.2)	238 (27.9)	
Body height, cm	161.0 ± 8.8	159.0 ± 9.5	<0.001[a]
<150, n (%)	835 (9.9)	143 (16.8)	<0.001[b]
150−159, n (%)	2,870 (34.0)	280 (32.9)	
160−169, n (%)	3,055 (36.2)	286 (33.6)	
≥170, n (%)	1,675 (19.9)	143 (16.8)	

AMD, age-related macular degeneration.

Data are presented as mean ± standard deviation or frequency (%).

[a]Wilcoxon rank-sum test was used for continuous variables.

[b]Chi-square test was used for categorical data.
