# Supplementary figures and images for: Adult body height and age-related macular degeneration in healthy individuals: A nationwide population-based survey from Korea
Source: PLoS One. 2020 May 1;15(5):e0232593. doi: 10.1371/journal.pone.0232593 (PMC7194362; doi:10.1371/journal.pone.0232593)

## Participants with AMD

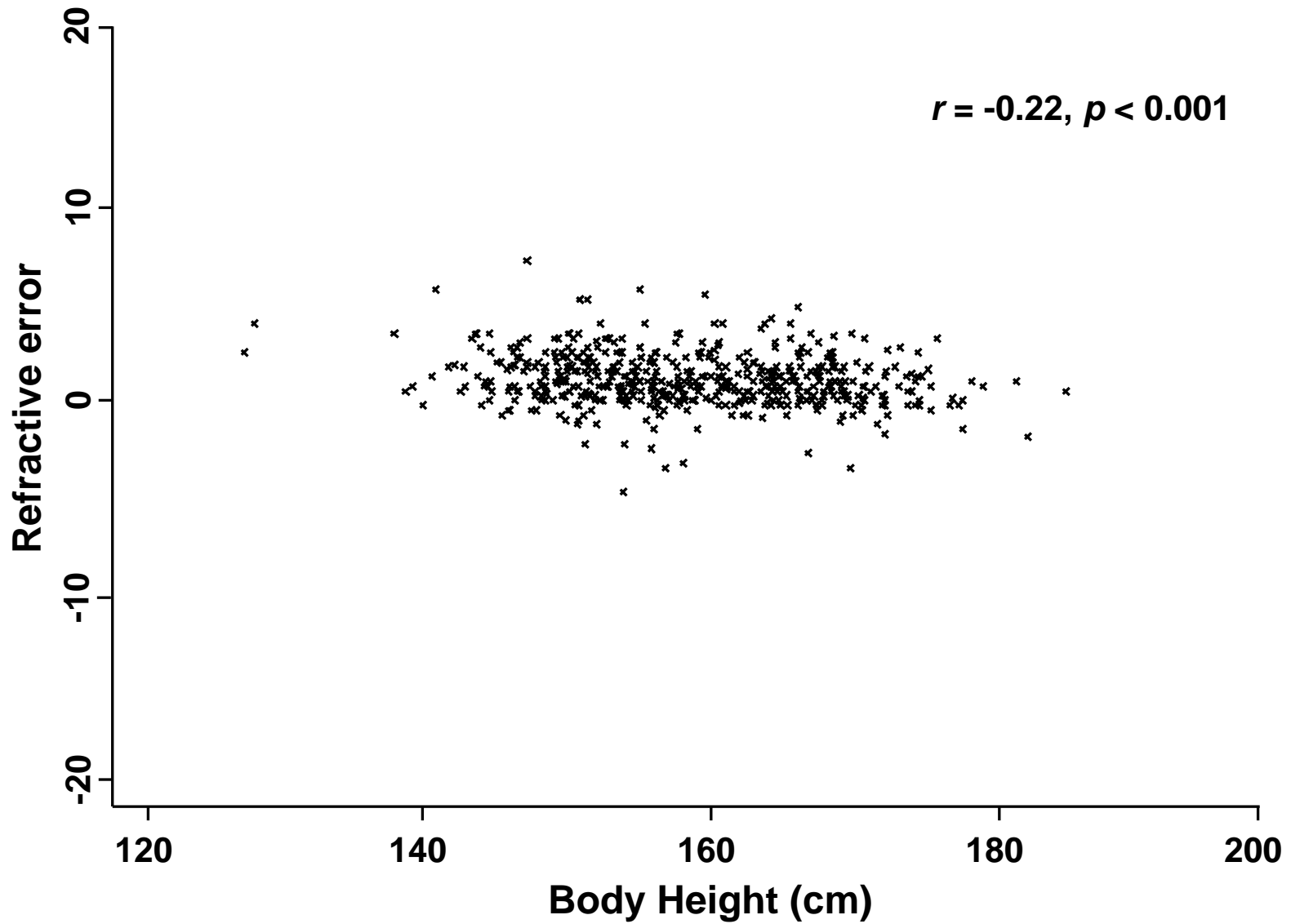

## Participants without AMD

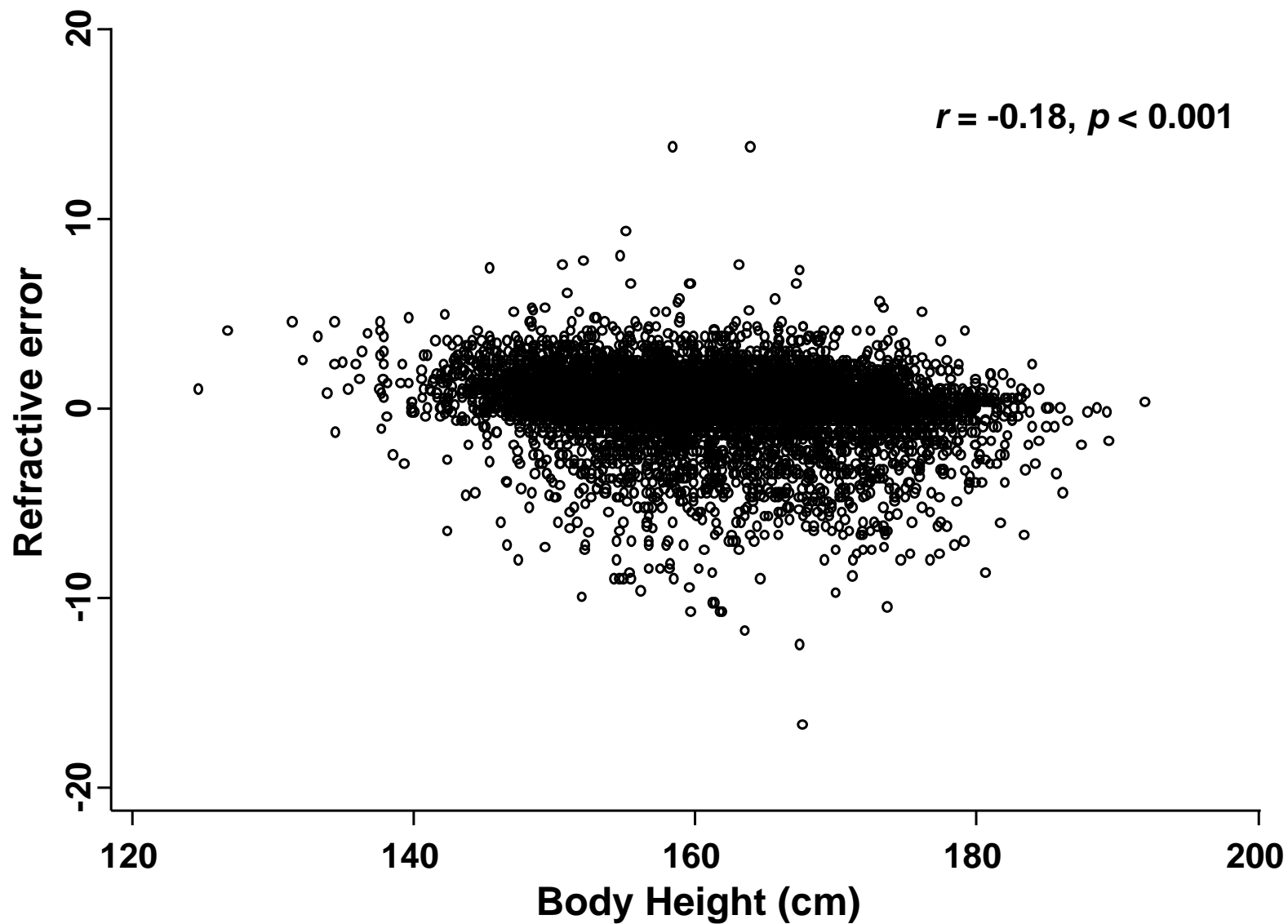

Supplement: S1 Fig — (PDF) [file pone.0232593.s002.pdf]
